# Campus Green Spaces, Academic Achievement and Mental Health of College Students

**DOI:** 10.3390/ijerph19148618

**Published:** 2022-07-15

**Authors:** Wanting Liu, Ning Sun, Jingyu Guo, Zhenhua Zheng

**Affiliations:** College of Communication and Art Design, University of Shanghai for Science and Technology, No. 516, Jungong Road, Shanghai 200093, China; 213513021@st.usst.edu.cn (W.L.); 1820180208@st.usst.edu.cn (N.S.); 213513012@st.usst.edu.cn (J.G.)

**Keywords:** college students, mental health, gender differences, academic achievement, campus green spaces

## Abstract

Mental health concerns have become a common problem among college students. Studies have shown that college students have a higher depression rate than the general population. The effect of campus green spaces on college students’ mental health has been widely studied. However, the internal mechanism of campus green spaces affecting college students’ mental health is not fully discussed. The data came from a cross-sectional survey of 45 Chinese universities. This paper discussed the relationship between campus green spaces, academic achievement, and college students’ mental health. Depending on gender, college students displayed different levels of mental health. The prevalence of depression among female students was higher than among male students. The effect of campus green spaces on mental health was higher in males than females, while the effect of campus green spaces on academic achievement had little gender difference. We call for the construction, improvement, and renewal of campus green spaces in the future not only to meet the needs of different gender groups, but also to pay more attention to the needs of female college students and improve the differences in mental health, so as to improve the mental health of the whole college student population.

## 1. Introduction

The World Health Organization lists depression and mental health problems as the leading causes of disability globally [1]. College students’ mental health receives extensive attention from society. College students are expected to have the best mental health and happiness [2]. However, studies have shown that college students have a higher depression rate than the general population [3]. College students face various social, academic, interpersonal, and environmental pressures, and a lack of psychological recovery may lead to depression and mental disorders [2,4,5,6]. With the rapid development of Chinese higher education, Chinese college students face fierce competition and employment pressure, which triggers a series of mental health problems [7,8].

Scholars of many disciplines have studied the relationship between mental health and nature, such as environmental psychology, geography, urban planning, medicine, and landscape architecture [9]. There is also a correlation between green perception and mental health [10,11]. There is a significant positive correlation between the quality of green spaces and better mental health [12]. The presence of greenery promotes psychological well-being and reduces depression and stress [13,14]. As an essential part of campus spaces, green spaces greatly affect students’ physical and mental recovery. Campus green spaces can significantly improve students’ mental health and reduce psychological pressure [5]. Numerous studies have demonstrated the importance of campus green spaces for students’ mental health [15,16]. Campus green spaces provide students with a way to relieve frustration and reduce stress [17].

Studies have shown that outdoor campus green spaces are considered a potential recovery environment, which helps students’ psychological recovery [18,19] and attention recovery [20]. Green spaces may explain their influence on academic achievement and mental health through two existing theories in the current research background: Attention Recovery Theory (ART) [21] and Stress Recovery Theory (SRT) [14]. According to ART, students recover their ability to actively guide attention through contact with the campus green space so as to restore their overall ability to learn and perform academic tasks [22]. Consequently, students’ academic achievement is improved. SRT proposes that students can achieve the effect of relieving stress through biological reactions to specific attributes of the natural environment after contacting campus green spaces [23]. These two theories show that campus green spaces can influence college students’ mental health positively.

Students’ success depends mainly on their academic achievement, so they have high expectations for their studies [24]. More academic stress often leads to increased depression [4]. Many studies have confirmed that mental health is strongly linked to academic achievement [25,26,27,28]. Campus green spaces are an external factor that affects college students’ academic achievement [29]. According to studies, increasing campus green spaces can improve students’ academic achievement [30]. Social ecology emphasizes that the effect of the environment on human health is not independent, but is affected by some mediating effects. The effect of campus green spaces on college students’ mental health is also not isolated and is jointly affected by multidimensional factors. For the special group of college students, academic achievement may be an important mediating variable between campus green spaces and the mental health of college students.

Previous studies have focused on the relationship between campus green spaces and college students’ mental health [6,15,31,32,33,34] and the relationship between academic achievement and mental health [8,35,36]. However, few studies have used academic achievement as a mediating variable. Meanwhile, the complex relationships between campus environment, achievement, and college students’ mental health have not been fully discussed.

At the same time, some scholars have paid attention to the gender differences in college students’ mental health. However, few studies have considered the reasons for gender differences in college students’ mental health. Our study focused on campus green spaces and the mental health of Chinese college students, explored whether there was a correlation between campus green spaces, academic achievement, and mental health of college students, and analyzed the differences between genders. Our research mainly addressed the following research questions:

1. Are there gender differences between Chinese college students’ mental health, campus green spaces, and academic achievement?
2. Do campus green spaces significantly affect college students’ mental health levels and academic achievement? Does academic achievement, the intermediary variable of campus green spaces, affect the mental health of college students?
3. Are there gender differences in the effects of campus green spaces and academic achievement on college students’ mental health status?

## 2. Materials and Methods

### 2.1. Study Population

The data came from a cross-sectional survey of 45 Chinese universities. The survey covered 45 universities, 20 provinces, and 30 cities in China. We obtained universal relevant data from Chinese universities by conducting an electronic questionnaire survey of students in the 45 universities in China. The questionnaire star survey platform (https://www.wjx.cn/app/survey.aspx, accessed on 9 November 2021) was our electronic questionnaire release. The survey was conducted under the supervision of the Academic Council of Shanghai University of Technology from 1 October 2021 to 30 January 2022. Furthermore, each college student participant completed the questionnaire voluntarily. Since the questionnaire star was an open questionnaire platform, to ensure the reliability of the data, the survey of this questionnaire was assisted by the deans, teachers, and other leaders of each university and college. In order to ensure the validity of the data, we set a password for the survey site and the login certificate and students cannot enter the certificate without the password. Students who participated in the survey were asked to answer specific related questions about the evaluation of campus green spaces, their mental health, and academic achievement. Eventually, 1261 students participated in the survey. With the effective organization of university leaders, the validity of the recovered questionnaire was as high as 98%, and 1236 valid samples were finally obtained. (The sample statistics see Table 1).

### 2.2. Measurement

#### 2.2.1. Dependent Variable: Mental Health of College Students

Our study evaluated the mental health of college students by self-rating. According to previous studies, depression assessment became the most important indicator of mental health. The Chinese version of the World Health Organization Five Happiness Index (WHO-5) evaluated depression in China. It included five positive emotional items: (1) feeling happy and comfortable; (2) feeling calm and relaxed; (3) feeling energized; (4) waking up feeling awake and well-rested, and; (5) everyday life is full of exciting things. College students’ mental health was assessed by asking the frequency of these five positive emotions in the recent two weeks. A score ranged from 6 to 0. Less than 13 points indicated depression. College students’ mental health was measured on a scale of 1 to 6. The higher score indicated better mental health.

#### 2.2.2. Independent Variable: Campus Green Spaces

Campus green spaces were part of students’ overall life experience, and the effect of campus green spaces on college students has been confirmed [5,30]. The green space in our study referred to college students’ subjective perception of campus green space environment [37]. Our study did not consider the temporal and spatial changes of the human position [38,39] and did not involve human mobility. Campus green spaces in this paper included four aspects: green comfort, reasonable layout, beautiful scenery, and diverse plants. In each item, the response ranged from 1 to 5 (1 = completely, 2 = disagree, 3 = neutral, 4 = completely, 5 = agree), and the higher score indicated the higher the respondents’ recognition of all aspects of campus green spaces.

#### 2.2.3. Intermediary Variables: Academic Achievement

Most researchers measured students’ academic achievement around GPA [40]. The academic achievement in this paper was college students’ self-assessment of their academic achievement. The item was scored on a scale of 1 to 5 (1 = very bad, 2 = not so good, 3 = general, 4 = better, 5 = very good), with the higher score indicating the better academic achievement of the respondents.

#### 2.2.4. Control Variables

In the conceptual model of this paper, education, parental education level, and monthly expenditure were included as control variables. Education levels were assigned as follows: 1 = freshman; 2 = sophomore; 3 = junior; 4 = senior; 5 = Master1; 6 = Master2; 7 = Master3; 8 = 1st Year PhD student; 9 = 2nd Year PhD student; 10 = 3rd Year PhD student and above. The item of parental education level was scored on a scale 1 to 7 (1 = Elementary school and below, 2 = junior high school, 3 = senior high school, technical secondary school and technical school, 4 = junior college, 5 = bachelor, 6 = master, 7 = doctor. The item of monthly expenditure was scored on a scale 1 to 7 (1 = less than 1000 yuan, 2 = 1000–2000 yuan, 3 = 2000–3000 yuan, 4 = 3000–5000 yuan, 5 = 5000–8000 yuan, 6 = more than 12,000 yuan.

### 2.3. Statistical Analysis

This study discussed the relationship between campus green spaces, academic achievement, and college students’ mental health. We validated the multi-factor analysis of all measurement models in the conceptual model. The results showed that all the measurement models’ compositional reliability was greater than 0.6; the average variance extraction was greater than 0.5; the factor load of the observed variables was greater than 0.6; the reliability coefficient was greater than 0.36 [41], and all the measurement models had good reliability and validity. The results of model fitting demonstrated that the indexes (CFI > 0.90, TLI > 0.90, RMSER > 0.08) achieved the criteria, which showed that the model was fit.

## 3. Results

### 3.1. Descriptive Statistics

The descriptive statistics of variables in Table 2 show that the Personal Health Assessment of College Students is greater than 13 points. These data indicate that most college students have good mental health. In the Personal Health Assessment of College Students, all the observed variables show a higher male score. The incidence of depression in female college students is 30.9%, and that in male college students is 22.5%. The incidence of depression in male college students is lower than in female college students (Table 3). All observed variables in campus green spaces are higher in females than males. Average scores of all college students are higher than 3, with males higher than females. In the control variables, the average education level for college students is sophomore, the father’s education level is above senior high school, the mother’s education level is above junior high school, and the monthly expenditure of college students is 1000–2000 yuan or more.

### 3.2. Analysis Based on the Models of Full Sample

**Figure 1:**
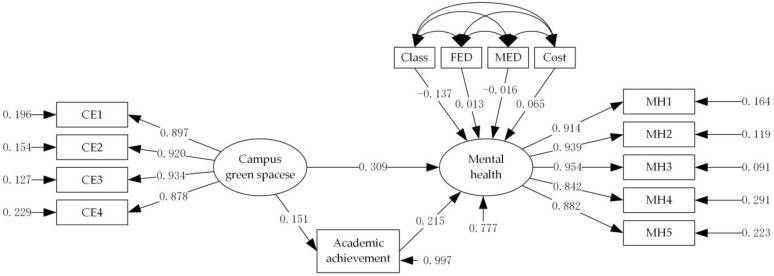
Standardized path diagram for the whole sample model.

Model-fitting results for the entire sample are shown in Table 4 and Figure 1. After controlling for education, parental education level, and monthly expenditure, the total effect values for campus green spaces and academic achievement on the mental health of college students are 0.341 and 0.215. The direct and indirect effects of campus green spaces on college students’ mental health are both significant, which indicate that this path may have intermediary variables. The intermediary effect value of academic achievement is 0.032. It indicates that the positive effect of campus green spaces on college students’ mental health should be realized by promoting academic achievement.

### 3.3. Comparison of Model Differences among Different Income Groups

**Figure 2:**
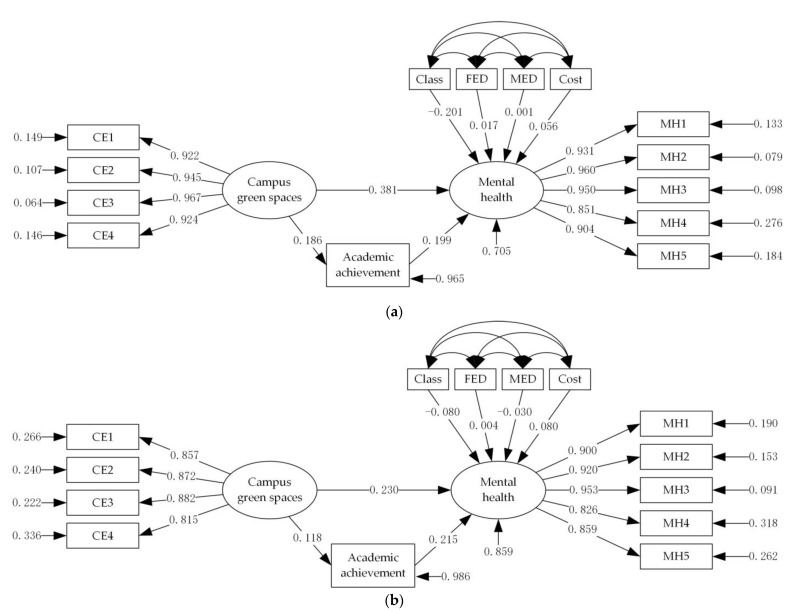
Comparison of standardized path diagram for different gender groups. (**a**) Male group. (**b**) Female group.

Our study compared college students of different gender groups to determine their model path. In the output results, the path coefficient was set to the same *p*-value < 0.05, indicating significant gender differences in the path of different genders college students group model. Table 5, Figure 2 compared the model-fitting results based on college students of different gender groups.

The mental health of male college students is significantly positively affected by campus green spaces and academic achievement. The total effect value of campus green spaces and academic achievement on the mental health of male college students are 0.418 and 0.199. The direct effect and the indirect effect of campus green spaces on college students’ mental health are significant, indicating that there is an intermediary effect within the path. Additionally, the intermediary effect value of academic achievement is 0.037. This result indicates that the positive effect of campus green spaces on male college students’ mental health needs to be realized by improving their academic achievement.

The mental health of female college students is significantly positively affected by campus green spaces and academic achievement, with effect values of 0.255 and 0.215. The direct effect and the indirect effect of campus green spaces on college students’ mental health are significant, indicating that there is an intermediary effect within the path. Additionally, the intermediary effect value of academic achievement is 0.025. This result indicates that the positive effect of campus green spaces on female college students’ mental health needs to be realized by improving their academic achievement.

## 4. Discussion

Our study explored the complex interaction between campus green spaces, academic achievement, and college students’ mental health. Moreover, our study found differences in college students’ mental health in relation to different genders. The mental health status of male college students was generally slightly higher than that of female college students. Male college students had a lower prevalence of depression than female college students, and the academic achievement of male college students was generally better than that of female college students.

Our study found that campus green spaces had an important effect on college students’ mental health, and the degree of this effect had exceeded that of academic achievement. This study evaluated campus green spaces from four aspects: greening comfort, reasonable layout, beautiful scenery, and diverse plants. Some studies confirmed that campus greening and natural contact could significantly improve college students’ mental health and reduce stress [5,6,42]. Therefore, this conclusion was consistent with the existing research conclusion.

Our study found that campus green spaces and academic achievement positively affected college students’ mental health. Meanwhile, academic achievement was the mediating variable of campus green spaces affecting college students’ mental health. The increase in campus green spaces positively affected academic achievement [30,43,44]. College students’ mental health was affected by academic achievement stress [25,26,27,28]. Campus green spaces affected the mental health of college students by affecting their academic achievement, and these three were the linkage effect. Therefore, to improve the mental health of college students, it was necessary to improve the quality of campus green spaces and academic achievement.

More importantly, our study found that campus green spaces and academic achievement significantly positively affected the mental health of college students of different genders. However, the degree of influence was different. The study found that the mental health of male college students was generally better than that of female college students, and the risk of depression of female college students is higher than that of male college students. For male college students, the effect of campus green spaces on mental health was greater than that of female students. There was a minor gender difference in the effect of campus green spaces on academic achievement, but there was no gender difference in the effect of academic achievement on college students’ mental health.

The influence of campus green spaces on the mental health of college students of different genders was different. Consequently, to improve the mental health of college students, it is necessary to consider the different needs of different genders. We need to put forward specific opinions and strategies according to the characteristics of different gender groups. First of all, campus green spaces positively affect college students’ mental health, and the improvement of campus green spaces is significant for both male and female college students. Meanwhile, the improvement of campus green spaces needs to start from various aspects. For male college students, campus green spaces have a more significant effect on their mental health. Therefore, improving campus green spaces can greatly improve the mental health of male college students. For female students, their mental health status is generally slightly lower than male students’. Therefore, female college students need special attention in campus groups. Their mental health is affected by both campus green spaces and academic achievement, and campus green spaces also affect academic achievement. Therefore, in campus green spaces construction, relevant decision-makers and environmental designers should give special attention to the requirement of female college students for campus green spaces: i) To improve female college students’ recognition of campus green spaces and relieve pressure, and; ii) To improve the effect of campus green spaces on female college students’ mental health, improve their academic achievement, and effectively improve the mental health of female college students from various aspects.

More specifically, the mental health of male college students is more affected by campus green spaces. Therefore, in the future improvement and renewal of campus green spaces, the mental health level of male college students can be improved by expanding the coverage area of the campus’s natural environment. However, the mental health of female college students is affected by both environment and performance. Consequently, in the future construction of the university landscape environment, we should pay more attention to campus green spaces that affect the performance of female college students and relieve their academic pressure. According to the ART theory, we can help female college students improve their academic achievement by creating more green spaces for rest and full contact with nature in the future campus green spaces improvement and renewal so as to improve the mental health of female college students. We call for the future improvement and renewal of campus green spaces, not only to meet the needs of different gender groups, but also to pay more attention to the needs of female college students, thereby reducing the differences in mental health and improving the mental health of the overall college student population.

Nevertheless, there are some limitations to the study. Firstly, the survey scope and the number of the sample are limited. This research could not represent all campus green spaces in China because only a few colleges are selected for an in-depth survey. In the future, more empirical studies need to be conducted. Furthermore, the representativeness of samples of college students must be improved. Finally, the assessment of campus green spaces is subjective. Our following research on the association between campus green spaces and the mental health of college students should combine subjective and objective assessments of campus green spaces assessments through more objective data, to better explore the relationship between campus green spaces and college students’ mental health.

## 5. Conclusions

Our findings found differences in mental health among Chinese college students. Female college students had slightly lower mental health status than male college students. The prevalence of depression was minimally higher than that of male college students. So, we need to pay special attention to the mental health of female college students.

Our study found that campus green spaces had an essential effect on college students’ mental health, and academic achievement was the mediating variable of campus green spaces affecting college students’ mental health. More importantly, we found the differences in the effects of campus green spaces on the mental health of college students of different genders. Campus green spaces had a more significant effect on the mental health of male college students, and there were minor gender differences in the influence of campus green spaces on academic achievement. There was no gender difference in the impact of performance on college students’ mental health.

The conclusions of this paper provide new ideas for the construction, improvement, and renewal of the current campus green spaces. For college students, campus green spaces, academic achievement, and mental health are social ecosystems that affect each other. In order to improve the overall mental health status of college students and reduce gender differences, it is necessary to put forward targeted strategies according to the characteristics of different genders of college students.

## Figures and Tables

**Table 1:** The sample demographics.

Demographics	N	%
Grade		
Freshman	440	35.6
sophomore	213	17.2
Junior	206	16.7
Senior	201	16.3
Master	158	12.7
Doctor	18	1.4
**Gender**		
Male	553	45.16
Female	683	54.84

**Table 2:** Variable descriptive statistics.

	Observed Variables	Variable Items	Mean Scores		
			Mean (All)	Mean (Male)	Mean (Female)
The mental health of college students (The personal mental health assessment)	**MH1**	In the recent two weeks, feeling happy and comfortable	4.451	4.529	4.386
	**MH2**	In the recent two weeks, feeling calm and relaxed	4.297	4.453	4.168
	**MH3**	In the recent two weeks, feeling energetic and energetic	4.298	4.416	4.201
	**MH4**	In the recent two weeks, feeling awake when you wake up and get enough rest	4.148	4.275	4.043
	**MH5**	In the recent two weeks, everyday life is full of interesting things	4.235	4.374	4.121
campus green spaces	**CE1**: Greening comfort	The greening area of our campus is large and comfortable.	3.800	3.671	3.907
	**CE2**: Reasonable layout	The green landscape layout of our campus is reasonable	3.728	3.630	3.809
	**CE3**: Beautiful scenery	The green landscape of our campus is beautiful	3.786	3.661	3.889
	**CE4**: Diverse plants	Our campus has a rich variety of plants and flowers	3.726	3.602	3.829
Academic achievement		How is your academic achievement	3.125	3.141	3.111
Controlvariable	educate	What education stage are you in now	2.728	2.372	3.022
	Parental education level	**FED**: What is your father’s academic degree	3.030	3.057	3.008
		**MED**: What is your mother’s academic degree	2.767	2.747	2.784
	Cost	How much do you spend every month	2.266	2.265	2.268

**Table 3:** The comparison of depression prevalence among different groups.

Gender	Depression Percentage%
female	30.9
male	22.5

**Table 4:** Total, direct and indirect effects of the full sample model.

Independent Variable	Intermediate Variable	Dependent Variable		
	Academic Achievement	Mental Health		
		Total Effect	Direct Effect	Indirect Effect
campus green spaces	0.151 ***	0.341 ***	0.309 ***	0.032 ***
Academic achievement	-	0.215 ***	0.215 ***	-

Note: *** means significant at the 1% confidence level.

**Table 5:** Comparison of the model paths in different gender groups.

Independent Variable		Intermediate Variable	Dependent Variable		
		Academic Achievement	Mental Health		
			Total Effect	Direct Effect	Indirect Effect
Male	campus green spaces	0.186 ***	0.418 ***	0.381 ***	0.037 ***
	Academic achievement	-	0.199 ***	0.199 ***	-
Female	campus green spaces	0.118 ***	0.255 ***	0.230 ***	0.025 ***
	Academic achievement	-	0.215 ***	0.215 ***	-

Note: *** means significant at the 1% confidence level.

## Data Availability

Data available on request due to restrictions privacy or ethical. The data presented in this study are available on request from the corresponding author.

## References

[1] World Health Organization Depression. Fact Sheets. https://www.who.int/news-room/fact-sheets/detail/depression.

[2] Eisenberg D., Gollust S.E., Golberstein E., Hefner J.L. (2007). Prevalence and correlates of depression, anxiety, and suicidality among university students. Am. J. Orthopsychiatry.

[3] Ibrahim A.K., Kelly S.J., Adams C.E., Glazebrook C. (2013). A systematic review of studies of depression prevalence in university students. J. Psychiatr. Res..

[4] Dyson R., Renk K. (2006). Freshmen adaptation to university life: Depressive symptoms, stress, and coping. J. Clin. Psychol..

[5] Kelz C., Evans G.W., Röderer K. (2013). The Restorative Effects of Redesigning the Schoolyard. Environ. Behav..

[6] Liu Q., Zhang Y., Lin Y., You D., Zhang W., Huang Q., van den Bosch C.C.K., Lan S. (2018). The relationship between self-rated naturalness of university green space and students’ restoration and health. Urban For. Urban Green..

[7] Hipp J.A., Gulwadi G.B., Alves S., Sequeira S. (2016). The Relationship Between Perceived Greenness and Perceived Restorativeness of University Campuses and Student-Reported Quality of Life. Environ. Behav..

[8] Sohail N. (2013). Stress and academic performance among medical students. J. Coll. Physicians Surg. Pak. JCPSP.

[9] Bratman G.N., Hamilton J.P., Daily G.C. (2012). The impacts of nature experience on human cognitive function and mental health. Ann. N. Y. Acad. Sci..

[10] Sugiyama T., Leslie E., Giles-Corti B., Owen N. (2008). Associations of neighbourhood greenness with physical and mental health: Do walking, social coherence and local social interaction explain the relationships?. J. Epidemiol. Community Health.

[11] Triguero-Mas M., Dadvand P., Cirach M., Martínez D., Medina A., Mompart A., Basagaña X., Gražulevičiene R., Nieuwenhuijsen M.J. (2015). Natural outdoor environments and mental and physical health: Relationships and mechanisms. Environ. Int..

[12] de Vries S., van Dillen S.M.E., Groenewegen P.P., Spreeuwenberg P. (2013). Streetscape greenery and health: Stress, social cohesion and physical activity as mediators. Soc. Sci. Med..

[13] Hartig T., Mang M., Evans G.W. (1991). Restorative Effects of Natural Environment Experiences. Environ. Behav..

[14] Ulrich R.S., Simons R.F., Losito B.D., Fiorito E., Miles M.A., Zelson M. (1991). Stress recovery during exposure to natural and urban environments. J. Environ. Psychol..

[15] Akpinar A. (2016). How is high school greenness related to students’ restoration and health?. Urban For. Urban Green..

[16] Yuan X., Zuo J., Huisingh D. (2013). Green Universities in China—What matters?. J. Clean. Prod..

[17] Lau S.S.Y., Yang F. (2009). Introducing Healing Gardens into a Compact University Campus: Design Natural Space to Create Healthy and Sustainable Campuses. Landsc. Res..

[18] Gulwadi G.B., Mishchenko E.D., Hallowell G., Alves S., Kennedy M. (2019). The restorative potential of a university campus: Objective greenness and student perceptions in Turkey and the United States. Landsc. Urban Plan..

[19] van den Bogerd N., Dijkstra S.C., Koole S.L., Seidell J.C., de Vries R., Maas J. (2020). Nature in the indoor and outdoor study environment and secondary and tertiary education students’ well-being, academic outcomes, and possible mediating pathways: A systematic review with recommendations for science and practice. Health Place.

[20] Lu M., Fu J. (2019). Attention Restoration Space on a University Campus: Exploring Restorative Campus Design Based on Environmental Preferences of Students. Int. J. Environ. Res. Public Health.

[21] Kaplan S., Berman M.G. (2010). Directed Attention as a Common Resource for Executive Functioning and Self-Regulation. Perspect. Psychol. Sci..

[22] Hodson C.B., Sander H.A. (2017). Green urban landscapes and school-level academic performance. Landsc. Urban Plan..

[23] Bowler D., Buyung-Ali L., Knight T.M., Pullin A.S. (2010). The importance of nature for health: Is there a specific benefit of contact with green space?. Systematic Review Collaboration for Environmental Evidence..

[24] Aghamolaei T., Shirazi M., Dadgaran I., Shahsavari H., Ghanbarnejad A. (2014). Health students’ expectations of the ideal educational environment: A qualitative research. J. Adv. Med. Educ. Prof..

[25] DeSocio J., Hootman J. (2004). Children’s Mental Health and School Success. J. Sch. Nurs..

[26] Hootman J., Houck G.M., King M.C. (2002). A Program to Educate School Nurses About Mental Health Interventions. J. Sch. Nurs..

[27] Lamb J.M., Puskar K.R., Sereika S., Patterson K., Kaufmann J.A. (2003). Anger Assessment In Rural High School Students. J. Sch. Nurs..

[28] Opie N.D., Slater P. (1988). Mental Health Needs of Children in School Role of the Child Psychiatric Mental Health Nurse. J. Child Adolesc. Psychiatr. Nurs..

[29] Fadilah Priyanda R., Amalia R. (2021). Analysis of external factors affecting students’ achievement student of mathematics education of samudra university. J. Phys. Conf. Ser..

[30] Matsuoka R.H. (2010). Student performance and high school landscapes: Examining the links. Landsc. Urban Plan..

[31] Fink J.E. (2014). Flourishing: Exploring Predictors of Mental Health within the College Environment. J. Am. Coll. Health.

[32] Malekinezhad F., Courtney P., bin Lamit H., Vigani M. (2020). Investigating the Mental Health Impacts of University Campus Green Space through Perceived Sensory Dimensions and the Mediation Effects of Perceived Restorativeness on Restoration Experience. Front. Public Health.

[33] McDonald-Yale E., Birchall S.J. (2021). The built environment in a winter climate: Improving university campus design for student wellbeing. Landsc. Res..

[34] Ibes D.C., Forestell C.A. (2020). The role of campus greenspace and meditation on college students’ mood disturbance. J. Am. Coll. Health..

[35] Rubach C., von Keyserlingk L., Simpkins S.D., Eccles J.S. (2022). Does Instructional Quality Impact Male and Female University Students Differently? Focusing on Academic Stress, Academic Satisfaction, and Mental Health Impairment. Front. Educ..

[36] Puskar K.R., Marie Bernardo L. (2007). Mental Health and Academic Achievement: Role of School Nurses. J. Spec. Pediatric Nurs..

[37] Barbosa O., Tratalos J.A., Armsworth P.R., Davies R.G., Fuller R.A., Johnson P., Gaston K.J. (2007). Who benefits from access to green space? A case study from Sheffield, UK. Landsc. Urban Plan..

[38] Chen B., Song Y., Jiang T., Chen Z., Huang B., Xu B. (2018). Real-Time Estimation of Population Exposure to PM2.5 Using Mobile- and Station-Based Big Data. Int. J. Environ. Res. Public Health.

[39] Song Y., Huang B., Cai J., Chen B. (2018). Dynamic assessments of population exposure to urban greenspace using multi-source big data. Sci. Total Environ..

[40] Darling-Hammond L., Rustique-Forrester E. (2005). The Consequences of Student Testing for Teaching and Teacher Quality. Teach. Coll. Rec. Voice Scholarsh. Educ..

[41] Fornell C., Larcker D.F. (1981). Evaluating Structural Equation Models with Unobservable Variables and Measurement Error. J. Mark. Res..

[42] Mcsweeney J., Rainham D., Johnson S.A., Sherry S.B., Singleton J. (2014). Indoor nature exposure (INE): A health-promotion framework. Health Promot. Int..

[43] Kuo M., Barnes M., Jordan C. (2019). Do Experiences with Nature Promote Learning? Converging Evidence of a Cause-and-Effect Relationship. Front. Psychol..

[44] Williams D.R., Dixon P.S. (2013). Impact of Garden-Based Learning on Academic Outcomes in Schools. Rev. Educ. Res..

